# Diagnosis & Treatment Options in Depression with comorbid Dementia

**DOI:** 10.1192/j.eurpsy.2024.93

**Published:** 2024-08-27

**Authors:** A. Young

**Affiliations:** Psychological Medicine, King’s College London, Institute of Psychiatry, Psychology & Neuroscience, London, United Kingdom

## Abstract

**Abstract:**

Depression and Dementia Professor Allan H Young, Head of Academic Psychiatry, Maudsley Hospital and King’s College London UK. allan.young@kcl.ac.uk

Mood Disorders are common, encompass cognitive impairments and occur in later life including first onset after the age of 50 years of age. There is a considerable overlap between depression and dementia. The relationship between depression and dementia will be reviewed and the implications for diagnosis and treatment will be discussed. Novel agents targeting alternative neurotransmitter pathways and inflammatory processes are promising potential treatment options. Neurostimulation treatments play a role with ECT at present having the best utility for late onset depression.

Key words: depression, dementia, antidepressants; pharmacotherapy

**Disclosure of Interest:**

A. Young Grant / Research support from: Principal Investigator in the Restore-Life VNS registry study funded by LivaNova. Principal Investigator on ESKETINTRD3004: “An Open-label, Long-term, Safety and Efficacy Study of Intranasal Esketamine in Treatment-resistant Depression.” Principal Investigator on “The Effects of Psilocybin on Cognitive Function in Healthy Participants” Principal Investigator on “The Safety and Efficacy of Psilocybin in Participants with Treatment-Resistant Depression (P-TRD)” Principal Investigator on “A Double-Blind, Randomized, Parallel-Group Study with Quetiapine Extended Release as Comparator to Evaluate the Efficacy and Safety of Seltorexant 20 mg as Adjunctive Therapy to Antidepressants in Adult and Elderly Patients with Major Depressive Disorder with Insomnia Symptoms Who Have Responded Inadequately to Antidepressant Therapy.” (Janssen) Principal Investigator on “ An Open-label, Long-term, Safety and Efficacy Study of Aticaprant as Adjunctive Therapy in Adult and Elderly Participants with Major Depressive Disorder (MDD).” (Janssen) Principal Investigator on “A Randomized, Double-blind, Multicentre, Parallel-group, Placebo-controlled Study to Evaluate the Efficacy, Safety, and Tolerability of Aticaprant 10 mg as Adjunctive Therapy in Adult Participants with Major Depressive Disorder (MDD) with Moderate-to-severe Anhedonia and Inadequate Response to Current Antidepressant Therapy.” Principal Investigator on “ A Study of Disease Characteristics and Real-life Standard of Care Effectiveness in Patients with Major Depressive Disorder (MDD) With Anhedonia and Inadequate Response to Current Antidepressant Therapy Including an SSRI or SNR.” (Janssen) UK Chief Investigator for Compass; COMP006 & COMP007 studies UK Chief Investigator for Novartis MDD study MIJ821A12201, Consultant of: Paid lectures and advisory boards for the following companies with drugs used in affective and related disorders: Flow Neuroscience, Novartis, Roche, Janssen, Takeda, Noema pharma, Compass, Astrazenaca, Boehringer Ingelheim, Eli Lilly, LivaNova, Lundbeck, Sunovion, Servier, Livanova, Janssen, Allegan, Bionomics, Sumitomo Dainippon Pharma, Sage, Neurocentrx

